# Factors Influencing Performance of Internet-Based Biosurveillance Systems Used in Epidemic Intelligence for Early Detection of Infectious Diseases Outbreaks

**DOI:** 10.1371/journal.pone.0090536

**Published:** 2014-03-05

**Authors:** Philippe Barboza, Laetitia Vaillant, Yann Le Strat, David M. Hartley, Noele P. Nelson, Abla Mawudeku, Lawrence C. Madoff, Jens P. Linge, Nigel Collier, John S. Brownstein, Pascal Astagneau

**Affiliations:** 1 International Department, French Institute for Public Health Surveillance (Institut de Veille Sanitaire), Saint Maurice, France; 2 Infectious Department, French Institute for Public Health Surveillance (Institut de Veille Sanitaire), Saint Maurice, France; 3 Department of Microbiology and Immunology, Georgetown University Medical Center, Washington, D.C, United States of America; 4 Imaging Science and Information Systems Center, Georgetown University School of Medicine, Washington, D.C, United States of America; 5 Department of Pediatrics, Georgetown University Medical Center, Washington, D.C, United States of America; 6 Centre for Emergency Preparedness and Response, Public Health Agency of Canada, Ottawa, Canada; 7 ProMED-mail, International Society for Infectious Diseases, Boston, Massachusetts, United States of America; 8 Joint Research Centre of the European Commission, Ispra, Italy; 9 National Institute of Informatics, Tokyo, Japan; 10 The European Bioinformatics Institute, Cambridge, United Kingdom; 11 Boston Children’s Hospital, Harvard Medical School, Boston, Massachusetts, United States of America; 12 École des Hautes Études en Santé Publique (EHESP), University school of public Health, PRES Sorbonne Cité, Paris, France; California Department of Public Health, United States of America

## Abstract

**Background:**

Internet-based biosurveillance systems have been developed to detect health threats using information available on the Internet, but system performance has not been assessed relative to end-user needs and perspectives.

**Method and Findings:**

Infectious disease events from the French Institute for Public Health Surveillance (InVS) weekly international epidemiological bulletin published in 2010 were used to construct the gold-standard official dataset. Data from six biosurveillance systems were used to detect raw signals (infectious disease events from informal Internet sources): Argus, BioCaster, GPHIN, HealthMap, MedISys and ProMED-mail. Crude detection rates (C-DR), crude sensitivity rates (C-Se) and intrinsic sensitivity rates (I-Se) were calculated from multivariable regressions to evaluate the systems’ performance (events detected compared to the gold-standard) 472 raw signals (Internet disease reports) related to the 86 events included in the gold-standard data set were retrieved from the six systems. 84 events were detected before their publication in the gold-standard. The type of sources utilised by the systems varied significantly (p<0001). I-Se varied significantly from 43% to 71% (p = 0001) whereas other indicators were similar (C-DR: p = 020; C-Se, p = 013). I-Se was significantly associated with individual systems, types of system, languages, regions of occurrence, and types of infectious disease. Conversely, no statistical difference of C-DR was observed after adjustment for other variables.

**Conclusion:**

Although differences could result from a biosurveillance system's conceptual design, findings suggest that the combined expertise amongst systems enhances early detection performance for detection of infectious diseases. While all systems showed similar early detection performance, systems including human moderation were found to have a 53% higher I-Se (p = 00001) after adjustment for other variables. Overall, the use of moderation, sources, languages, regions of occurrence, and types of cases were found to influence system performance.

## Introduction

Emerging and re-emerging infectious diseases continue to pose major threats to global health security [1], [2]. The Internet provides information that can be used to detect health threats early [3]. Epidemic intelligence (EI) relies mainly on *event-based* biosurveillance, i.e. the *ad hoc* detection and interpretation of unstructured information originating from multiple and not predefined sources on the Internet. Sources are varied but typically include the electronic news media and official governmental and non-governmental organisations [4]. Internet-based biosurveillance systems have been developed to monitor this large volume of information [5]. Despite substantial inherent differences, these systems all scan the Internet to detect reported related to infectious disease that could represent potential health threats, and filter unstructured information through complex algorithms. Select relevant information is stored on dedicated web-based platforms and disseminated. Information collected is then further filtered, verified, and analysed by end-users (i.e. national or international institution and stakeholders involved in EI management).

One of the limitations of event-based biosurveillance is the difficulty of applying traditional epidemiological parameters (e.g. sensitivity, specificity, positive predictive value, etc.), due to the lack of accessible data on verified outbreaks (i.e. a gold standard). The use of Internet-based biosurveillance systems is still maturing and its assessment is on-going [6], [7]. Most of the available scientific literature focuses either on the assessment of biosurveillance system performance regarding detection and adequate classification of health-related information using informal open sources, or on the presentation of innovative functionalities. An important topic yet to be elucidated in the literature concerns the performance of biosurveillance systems relative to end-user needs and expectations.

This study aims at providing a quantitative evaluation of multiple biosurveillance systems’ performance compared to a gold-standard.

## Methods

### Epidemic intelligence in France

France is a medium sized country made up of metropolitan France and eleven overseas territories scattered over Africa, America, and Oceania. French surveillance has been focused traditionally on the detection of unusual health events occurring in the national territory [8], [9]. In 2002 the International Department of the French institute for public health surveillance (InVS) developed EI to detect internationally emerging health threats that could affect the French population living in France and abroad [10]. The process was formalized into five steps: detection of informal disease reports (e.g. using biosurveillance systems), selection of disease events (through a set of defined criteria), validation of the event (through a network of contacts, available official information, etc.), analysis, and communication [11].

Events targeted to the InVS public health network are integrated into the weekly international epidemiological bulletin (BHI) available on the InVS website every Wednesday [12]. Only verified events are reported in the BHI, and events are usually reported only once. Updates can occasionally be integrated but only if major epidemiological changes occur.

### Definitions

#### Events

Events were defined as a verified infectious disease occurrence resulting from the EI process (i.e. including verification and analysis). Events were defined by the disease, the type of cases (human or animal), the country, the province or state, and the month of occurrence regardless of the number of cases concerned. All events are considered to be independent.

#### Signals

Signals were defined as unverified raw infectious disease information (in relation to an event included in the gold-standard data set) collected from biosurveillance systems (informal sources). Biosurveillance systems are therefore a source of signal, but are not the source of the events included in the BHI (i.e. gold-standard data set).

Biosurveillance systems are used only for the detection of signals. Once selected signals are fully processed (i.e. systematically verified, analysed, characterised, etc.), signals can be classified as an “event” or “discarded”. If biosurveillance systems are the main sources of signals, in the EI process they are not the “source of the event”.

#### Gold-Standard

Ideally, infectious disease signals identified by Internet biosurveillance methods should be compared to official event reports (gold-standard) during the same time period. The EI performed at InVS, for which necessary information (rational, selection and validation processes, etc.) was readily accessible, was chosen as the best source of gold-standard data for this study.

Infectious disease events (human cases and zoonosis epizootics) reported in the BHI in 2010 were included in the gold-standard data set. A/H5N1 influenza has been considered a health threat for a long time though it is subject to substantial under-reporting [13]. Hence, A/H5N1 cases were excluded from the study. Non-infectious disease events, not systematically monitored by all systems, were also excluded from our study.

#### Biosurveillance Systems

Six biosurveillance systems contributing to the Early Alerting and Reporting (EAR) project launched under the Global Health Security Initiative (GHSI) [14] were used to detect raw signals (informal reports of infectious disease): Argus, BioCaster, GPHIN, HealthMap, MedISys and ProMED (Table 1). Of those, three are fully moderated (i.e. include human analysts in the selection, sorting and/or translation processes) while the remaining three systems are based mainly on automated processes for detection, sorting, and translation (Table 1).

**Table 1:** Biosurveillance systems included in the study.

System name	System owner/developer	Country	Starting date	Type of Moderation	n languages	references
Argus	Georgetown University Medical Center	USA	2004	Human moderation	50	[22]
BioCaster	National Institute of Informatics	Japan	2006	Fully automated	7	[23]
GPHIN	Public Health Agency of Canada	Canada	1997	Human moderation	9	[24]
HealthMap	Harvard University	USA	2006	Automated*	7	[25]
MedISys	Joint Research Centre	EU	2004	Fully automated	60	[26]
ProMED	International Society of Infectious Diseases	USA	1994	Human moderation	7	[27]

*Partially moderated.

#### Data

Signals relating to events included in the gold-standard data set were retrospectively searched on all six biosurveillance systems through *ad hoc* queries using keywords or a series of keywords. Searches were performed by two InVS epidemiologists (i.e. independent from the six biosurveillance systems). Discordant pairs were reviewed and the most relevant signal was kept in the final database.

#### Rates

The crude detection rate (C-DR) was defined as the ability of a system to detect an infectious disease event, the intrinsic detection rate (I-DR) was defined as the ability of a system to detect outbreaks independently from other systems. The crude sensitivity rate (C-Se) was defined as the ability of a system to detect outbreaks prior to their publication in the BHI. The intrinsic sensitivity rate (I-Se) was defined as the ability of a system to detect outbreaks independently from other systems and before their publication in the BHI (see table 2 for details).

**Table 2:** Definition of indicators and rates.

Indicators	Abrev.	Definition
Crude Detected Event	CDET	First signal relating to a health event included in the gold-standard
Intrinsically Detected Event	IDET	First signal detected primarily by the system (excluding signal originating from another system included in the study)
Not-Detected Event	NDET_1_	Event not detected by the system
	NDET_2_	Event not detected by the system or not primarily detected by the system
Crude True Positive event	CTP	First signal related to an event included in the gold-standard and detected by a system before the reporting of the event in the BHI
Intrinsic True Positive event	ITP	First signal related to an event included in the gold-standard, detected by a system before its reporting in the BHI and primarily detected by the system
False Negative	FN_1_	Event not detected by the system before its reporting in the BHI
	FN_2_	Event not detected by the system before its reporting in the BHI or not detected primarily by the system
**Rates**	**Abrev.**	**Definition**
Crude Detection Rate	C-DR	Ability of a system to detect confirmed infectious disease outbreaks. C-DR = (C-DET)/(CDET+NDET_1_)
Intrinsic Detection Rate	I-DR	Ability of a system to detect confirmed infectious disease outbreaks independently from other systems. I-DR = (IDET+NDET_2_)
Crude Sensitivity Rate	C-Se	Ability of a system to detect confirmed infectious disease outbreaks prior to the publication in the BHI. C-Se = (CTP)/(CTP+FN_1_).
Intrinsic Sensitivity Rate	I-Se	Ability of a system to detect confirmed infectious disease outbreaks independently from other systems and before their publication in the BHI. I-Se = (ITP)/(ITP+FN_2_)).

#### Associated factors

Bivariable and multivariable modified Poisson regressions (well suited to produce rate ratios) were used to assess the associated factors [15]. Rate Ratios (RR), 95% confidence intervals (CI) and p values were computed to assess the strength of these associations. The same variables were included in the different regression models. Potential interactions were tested for their significance at the 005 level. All analyses were performed using Stata 12.1 (StataCorp LP, USA).

## Results

### Gold-Standard

**Figure 1:**
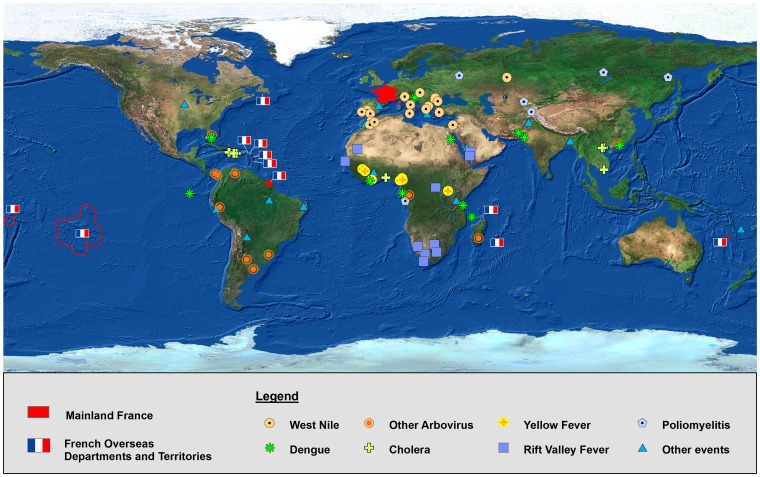
Type and geographical distribution of events published in the BHI in 2010.

In 2010, 132 events were reported in the BHI. 46 (35%) were excluded (26 global overviews or long-lasting events, 12 follow-ups of previously reported events and 8 non-infectious health occurrences). The 86 events included in the gold-standard data set occurred in 46 countries; 23 (27%) in the Americas, 22 (25%) in Africa, 17 (20%) in Europe, 14 (16%) in Asia and 10 (12%) in Near-East and North-Africa (Figure 1). Twenty-two disease events were identified. West Nile virus (WNV) infection and dengue infections represented 21% (n = 18) and 14% (n = 12) of the events, respectively. Important variations were observed according to the region of occurrence, e.g. WNV infection represented 76% of events reported in Europe, 50% in the North-Africa and Near-East, but none of the events reported in America or in Asia (Table 3).

**Table 3:** Nature and geographic distribution of events reported in InVS weekly international bulletin (BHI), 2010.

Diseases	America		Sub-Saharan Africa		Asia		Europe			North-Africa Middle-East		Total	
	n	*%*	n	%	n	%	n	%	n		%	n	%
West Nile	-	*-*	-	*-*	-	*-*	13	*(76%)*	5		*(50%)*	18	*(21%)*
Dengue	4	*(17%)*	4	*(18%)*	2	*(14%)*	1	*(6%)*	1		*(10%)*	12	*(14%)*
Cholera	5	*(22%)*	1	*(5%)*	4	*(29%)*	-	*-*	-		*-*	10	*(12%)*
Rift Valley Fever	-	*-*	6	*(27%)*	-	*-*	-	*-*	2		*(20%)*	8	*(9%)*
Yellow fever	-	*-*	6	*(27%)*	-	*-*	-	*-*	-		*-*	6	*(7%)*
Poliomyelitis	-	*-*	1	*(5%)*	4	*(29%)*	1	*(6%)*	-		*-*	6	*(7%)*
Chikungunya	-	*-*	2	*(9%)*	1	*(7%)*	-	*-*	-		*-*	3	*(3%)*
Plague	2	*(9%)*	1	*(5%)*	-	*-*	-	*-*	-		*-*	3	*(3%)*
Malaria	-	*-*	-	*-*	-	*-*	2	*(12%)*	1		*(10%)*	3	*(3%)*
Saint Louis enc.	2	*(9%)*	-	*-*	-	*-*	-	*-*	-		*-*	2	*(2%)*
Mayaro	2	*(9%)*	-	*-*	-	*-*	-	*-*	-		*-*	2	*(2%)*
Measles	2	*(9%)*	-	*-*	-	*-*	-	*-*	-		*-*	2	*(2%)*
Venezuelan Eq. Enc.	2	*(9%)*	-	*-*	-	*-*	-	*-*	-		*-*	2	*(2%)*
Eastern Eq. Enc.	1	*(4%)*	-	*-*	-	*-*	-	*-*	-		*-*	1	*(1%)*
Oropuche	1	*(4%)*	-	*-*	-	*-*	-	*-*	-		*-*	1	*(1%)*
Crimean–Congo HF	-	*-*	-	*-*	1	*(7%)*	-	*-*	-		*-*	1	*(1%)*
Nipah	-	*-*	-	*-*	1	*(7%)*	-	*-*	-		*-*	1	*(1%)*
Alkhurma	-	*-*	-	*-*	-	*-*	-	*-*	1		*(10%)*	1	*(1%)*
Influenza	1	*(4%)*	-	*-*	-	*-*	-	*-*	-		*-*	1	*(1%)*
Typhoid	-	*-*	-	*-*	1	*(7%)*	-	*-*	-		*-*	1	*(1%)*
Diphtheria	1	*(4%)*	-	*-*	-	*-*	-	*-*	-		*-*	1	*(1%)*
Anthrax	-	*-*	1	*(5%)*	-	*-*	-	*-*	-		*-*	1	*(1%)*
Total	23	*(100%)*	22	*(100%)*	14	*(100%)*	17	*(100%)*	10		*(100%)*	86	*(100%)*

Eq. Enc.  =  Equine encephalitis HF  =  Haemorrhagic fever.

### Signals Detected

A total of 472 raw signals relating to events included in the gold-standard data set were retrieved from the six biosurveillance systems. The language of the initial source was English for 53% (n = 249), Spanish for 15% (n =  72), French for 11% (n = 53) and other languages for 20% (n = 94). The remaining 4 events were detected through several sources in different languages and information was insufficient to rank them. Language sources varied according to the systems (p = 0.063) and to the region of occurrence of the event (p<0.001). All 86 events included in the gold-standard data set were detected by at least one of the systems and 57 (66%) were detected by all six systems. When early detection was considered, 84 (98%) events were detected before their publication in the BHI, 30 (35%) were detected by the six systems, 26 (30%) by five systems, 10 (12%) by four systems, 7 (8%) by three systems, 6 (7%) by two systems, 5 (6%) by a single system and 2 events (2%) were not detected prior to their publication in the BHI. According to the system the proportion of media sources utilised by the systems ranged from 44% to 73%, official sources from 6% to 32%. Raw signals originating from another system included in the study ranged from 1% to 49% (p<0.001) (Table 4).

**Table 4:** Distribution Variables.

Types source	Argus		BioCaster		GPHIN		HealthMap		MedISys		ProMED		Total		p value
	n	%	n	%	n	%	n	%	n	%	n	%	n	%	
Media §	53	67%	35	44%	66	93%	40	49%	42	52%	58	73%	294	62%	
Systems *	1	1%	39	49%	1	1%	32	39%	33	41%	1	1%	107	23%	<0.001
Official #	25	32%	5	6%	4	6%	10	12%	6	7%	21	26%	71	15%	
Total	79	100%	79	100%	71	100%	82	100%	81	100%	80	100%	472	100%	

Languages	Argus		BioCaster		GPHIN		HealthMap		MedISys		ProMED		Total		p value
	n	%	n	%	n	%	n	%	n	%	n	%	n	%	
English	37	49%	46	49%	33	46%	39	48%	51	63%	43	54%	249	53%	
Spanish	7	9%	11	9%	12	17%	16	20%	8	10%	18	23%	72	15%	
French	9	12%	6	12%	11	15%	7	9%	8	10%	12	15%	53	11%	
Russian	7	9%	4	9%	4	6%	8	10%	4	5%	3	4%	30	6%	0.063
Portuguese	1	1%	4	1%	5	7%	7	9%	3	4%	2	3%	22	5%	
Other	14	19%	8	19%	6	8%	5	6%	7	9%	2	3%	42	9%	
Total	75	100%	79	100%	71	100%	82	100%	81	100%	80	100%	468	100%	

Regions of occurrence	Argus		BioCaster		GPHIN		HealthMap		MedISys		ProMED		Total		p value
	n	%	n	%	n	%	n	%	n	%	n	%	n	%	
Sub-Sah. Afr £	22	28%	21	27%	20	28%	21	26%	22	27%	22	28%	128	27%	
America	16	20%	21	27%	19	27%	22	27%	22	27%	22	28%	122	26%	
Europe	17	22%	15	19%	11	15%	17	21%	15	19%	16	20%	91	19%	1.00
Asia	14	18%	14	18%	14	20%	14	17%	14	17%	14	18%	84	18%	
N. Afr.- M.East ‡	10	13%	8	10%	7	10%	8	10%	8	10%	6	8%	47	10%	
Total	79	100%	79	100%	71	100%	82	100%	81	100%	80	100%	472	100%	

Types of disease	Argus		BioCaster		GPHIN		HealthMap		MedISys		ProMED		Total		p value
	n	%	n	%	n	%	n	%	n	%	n	%	n	%	
Encephalitis	23	29%	19	24%	16	23%	22	27%	19	23%	18	23%	117	25%	
Dengue-like	16	20%	21	27%	15	21%	20	24%	21	26%	20	25%	113	24%	
VHF ¥	7	9%	7	9%	7	10%	7	9%	7	9%	7	9%	42	9%	1.00
Diarrhoeal	10	13%	10	13%	11	15%	10	12%	10	12%	11	14%	62	13%	
Other	23	29%	22	28%	22	31%	23	28%	24	30%	24	30%	138	29%	
Total	79	100%	79	100%	71	100%	82	100%	81	100%	80	100%	472	100%	

Types of case	Argus		BioCaster		GPHIN		HealthMap		MedISys		ProMED		Total		p value
	n	%	n	%	n	%	n	%	n	%	n	%	n	%	
Human	64	81%	67	85%	61	86%	68	83%	70	86%	68	85%	398	84%	
Animal	15	19%	12	15%	10	14%	14	17%	11	14%	12	15%	74	16%	0.94
Total	79	100%	79	100%	71	100%	82	100%	81	100%	80	100%	472	100%	

Regions of occurrence	English		Spanish		French		Russian		Portuguese		Other		Total		p value
	n	%	n	%	n	%	n	%	n	%	n	%	n	%	
Sub-Sah. Afr£	75	30%	1	1%	44	83%	3	10%	1	5%	3	7%	127	27%	
America	51	20%	46	64%	2	4%	0	0%	20	91%	1	2%	120	26%	
Europe	52	21%	21	29%	1	2%	9	30%	1	5%	7	17%	91	19%	<0.001
Asia	45	18%	4	6%	1	2%	15	50%	0	0%	18	43%	83	18%	
N. Afr.- N.East ‡	26	10%	0	0%	5	9%	3	10%	0	0%	13	31%	47	10%	
Total	249	100%	72	100%	53	100%	30	100%	22	100%	42	100%	468	100%	

§ Media  =  Press, news aggregators and blogs.

* Systems  =  another biosurveillance system included in the study.

# Official  =  official sources and expert contributions.

£ Sub-Sah. Afr  =  Sub-Saharan Africa.

‡ N. Afr.- N.East  =  North Africa and Near-East.

¥ VHF  =  Viral Haemorrhagic Fever.

The systems’ C-DR ranged from 83% to 95% (p = 020), I-DR ranged from 47% to 92% (p<0.001), C-Se ranged from 71% to 85%% (p = 013), and I-Se ranged from 43% to 71% (p = 0001) (Table 5).

**Table 5:** Detection, sensitivity rates and rate ratios from bivariable Poisson regressions

		Argus	BioCaster	GPHIN	HealthMap	MedISys	ProMED	p value
Crude Detection	CDET	79	79	71	82	81	80	0.20
	C-DR	92%	92%	83%	95%	94%	93%	
	RR	-	1.00	0.90	1.04	1.03	1.01	
	CI	-	0.91–1.09	0.80–1.00	0.95–1.12	0.94–1.11	0.92–1.10	
	p value	-	1.00	0.07	0.35	0.55	0.77	
Intrinsic Detection	IDET	78	40	70	50	48	79	<0.001
	IDR	91%	47%	81%	58%	56%	92%	
	RR	1.11	0.57	-	0.71	0.69	1.13	
	CI	0.99–1.26	0.45–0.73	-	0.58–0.88	0.55–0.85	1.00–1.27	
	p value	0.08	<0.001	-	0.001	0.001	0.05	
Crude Sensitivity	CTP	61	63	62	73	67	62	0.13
	C-Se	71%	73%	72%	85%	78%	72%	
	RR	0.84	0.86	0.85	-	0.92	0.85	
	CI	0.71–0.98	0.73–1.00	0.72–0.99	-	0.79–1.05	0.72–0.99	
	p value	0.03	0.06	0.04	-	0.24	0.04	
Intrinsic Sensitivity	ITP	60	37	62	43	44	61	0.001
	I-Se	70%	43%	72%	50%	51%	71%	
	RR	0.98	0.61	1.02	0.70	0.72	-	
	CI	0.81–1.19	0.46–0.80	0.84–1.23	0.55–0.91	0.56–0.92	-	
	p value	0.87	<0.001	0.87	0.006	0.01	-	

RR =  Rate Ratio; CI =  95% confidence interval.

None of the system can be considered as a reference and similar results were found using different combinations. For the table, moderated systems were alternatively chosen (alphabetic order) as reference and HealthMap was selected as the reference for CTP to improve readability.

### Factors associated with Crude or Intrinsic detection

In the bivariable regression analysis individual systems, moderation of system, languages, regions of occurrence, types of disease, and types of case were significantly associated (Table 6). No significant differences were observed across and within systems for C-DR; I-DR and I-Se varied significantly across and within systems; one system (HealthMap) showed a significantly higher C-Se than moderated systems, but across systems the difference was not significant (p = 0.13) (Table 5).

**Table 6:** Factors associated with crude or intrinsic detection from bivariable Poisson regressions.

		Crude detection				Intrinsic detection			
		RR	95% CI	p value	p value	RR	95% CI	p value	p value
**Systems**	ProMED	-	-	-		-	-	-	
	Argus	0.98	0.81–1.19	0.87		0.98	0.81–1.19	0.87	
	BioCaster	1.02	0.85–1.22	0.86		0.61	0.46–0.80	0.00	
	Gphin	1.00	0.83–1.20	1.00	0.131	1.02	0.84–1.23	0.87	<0.001
	HealthMap	1.18	1.00–1.38	0.04		0.70	0.55–0.91	0.01	
	MedISys	1.08	0.91–1.29	0.38		0.72	0.56–0.92	0.01	
**Moderation of systems**	Automated	-	-	-	-	-	-	-	-
	Moderated	0.91	0.82–1.01	0.07	-	1.48	1.27–1.71	0.0001	-
**Types of diseases**	Encephalitis	-	-	-		-	-	-	
	Dengue-like	1.15	0.97–1.36	0.12		1.16	0.91–1.48	0.22	
	VHF ^¥^	1.45	1.23–1.71	<0.001	<0.001	1·69	1.33–2.15	<0.001	<0.001
	Diarrhoeal	1.39	1.18–1.63	<0.001		1.73	1.40–2.15	<0.001	
	Others	1.30	1.12–1.52	0.001		1.33	1.07–1.66	0.01	
**Regions of occurrence**	America	-	-	-		-	-	-	
	Sub-Sah. Afr £	1·16	1·02–1·33	0·03		1·33	1·09–1·63	0·01	
	Europe	0·90	0·75–1·08	0·24	<0·001	1·02	0·80–1·31	0·85	<0·001
	Asia	1·27	1·12–1·45	<0·001		1·57	1·29–1·91	<0·0001	
	N. Afr.-N.East ‡	0·94	0·76–1·16	0·55		0·92	0·67–1·26	0·61	
**Types of case**	Human	-	-	-	-	-	-	-	-
	Animal	0.72	0.59–0.86	<0.001	-	0.77	0.61–0.97	0.03	-
**Types of source**	Media §	-	-	-		-	-	-	-
	Systems *	0.86	0.77–0.97	0.01	0.001	-	-	-	-
	Official #	0.79	0.67–0.92	0.004		-	-	-	-

RR =  Rate Ratio; 95% CI =  95% confidence interval.

§ Media  =  Press, news aggregators and blogs.

* Systems  =  another biosurveillance system included in the study.

# Official  =  official and expert contributions.

£ Sub-Sah. Afr  =  Sub-Saharan Africa.

‡ N. Afr.- N.East  =  North Africa and Near-East.

¥ VHF  =  Viral Haemorrhagic Fever.

From the multivariable Poisson regression models, no statistical difference in C-Se was observed after adjustment for individual systems, moderation, languages, types of diseases, regions of occurrence, and type of cases. Conversely, systems, moderation, languages, regions of occurrence, and types of disease were all significantly associated with I-Se (Table 7). Potential interactions were tested, and none were found significant at the 0.05 level.

**Table 7:** Factors associated with crude or intrinsic detection from multivariable Poisson regression models.

		Crude detection			Intrinsic Detection		
	Variable	RR	95% CI	p value	RR	95% CI	p value
**Types of System**	Automated	-	-	-	-	-	-
	Moderated	0.96	0.88–1.04	0.27	1.53	1.34–1.75	<0.001
**Languages of detection**	English	-	-	-	-	-	-
	Spanish	1.10	0.96–1.27	0.18	1.22	0.99–1.51	0.06
	French	0.99	0.86–1.14	0.93	1.06	0.85–1.33	0.58
	Other	1.08	0.98–1.20	0.13	1.21	1.01–1.44	0.04
**Regions of occurrence**	America	-	-	-	-	-	-
	Sub-Sah. Afr £	1.10	0.95–1.28	0.20	1.25	0.98–1.60	0.07
	Europe	0.95	0.79–1.13	0.54	1.18	0.91–1.52	0.22
	Asia	1.09	0.97–1.24	0.15	1.30	1.07–1.59	0.01
	N. Afr.-N.East ‡	1.16	0.98–1.37	0.08	1.15	0.86–1.53	0.35
**Types of disease**	Encephalitis	-	-	-	-	-	-
	Dengue-like	0.95	0.80–1.12	0.53	1.11	0.84–1.47	0.46
	VHF ¥	1.06	0.86–1.31	0.60	1.39	0.99–1.94	0.06
	Diarrheal	1.09	0.91–1.30	0.34	1.55	1.16–2.06	0.003
	Other	1.01	0.85–1.21	0.90	1.14	0.87–1.49	0.33
**Types of cases**	Human	-	-	-	-	-	-
	Animal	0.84	0.68–1·02	0.08	1.08	0.84–1.37	0.55

RR =  Rate Ratio; 95% CI =  95% confidence interval.

£ Sub-Sah. Afr  =  Sub-Saharan Africa.

‡ N. Afr.- N.East  =  North Africa and Near-East.

¥ VHF  =  Viral Haemorrhagic Fever.

## Discussion

The systems’ characteristics (type of moderation, sources accessed, diseases, languages, and regions covered) were found to significantly influence disease detection performance. This highlights the differences in conceptual design used to develop the biosurveillance systems, and the importance of taking advantage of synergies through combining systems’ data for infectious diseases detection.

C-DR was used to evaluate the global detection rate independently from the EI quality and type. With C-DR values ranging from 83% to 95%, all systems were found to have a similar ability to detect infectious events, findings consistent with other studies [23-34]. However, C-DR is a very crude indicator that does not take into consideration the main EI objective of early detection.

C-Se provides a better estimation of the systems’ ability to detect infectious diseases outbreaks early in a given framework defined by the chosen gold-standard. Although biosurveillance systems were originally designed to detect relevant information though informal sources, they now include a noteworthy proportion of early released official information. C-Se of early published official information was 27% lower than C-Se of media sources (p = 0001) underlining the usefulness of media sources in the detection of communicable diseases outbreaks. Despite their different conceptual designs and notably the type of sources used, all systems demonstrated remarkably similar early detection capacities as C-Se remained comparable even after adjusting for other variables (p = 013).

A high level of cross-feeding (i.e., a system using another fellow system as a source) was documented, further emphasising the synergistic qualities of the systems. For automated systems, 39% of early detected signals were collected from another system versus 1% for moderated systems (p<0001). I-Se provides an appropriate proxy to assess a system’s detection rate. Individually (data not shown) and collectively (Table 6) moderated systems were found to have a 53% higher I-Se (p<0001) than automated systems after adjustment for the other variables included in the model. This increased I-Se of moderated systems can be attributed directly to their common characteristic: the human moderation. The difficulties met by the systems in developing an efficient algorithm covering the different facets of a single disease have been demonstrated by a previous study [7]. Hence, our study illustrates the significant added value resulting from the input of human analysts and their ability to balance the limits inherent to a fully automated detection. Yet, I-Se should be considered as a lower limit, because for each system only the first detected signal was considered. Therefore, it cannot be excluded that some signals primarily collected via a fellow system may have been later detected through another source, but still earlier than the gold-standard. All systems integrate a de-duplication module, aiming at reducing the volume of redundant information; de-duplication performance varies according to systems. A consistent collection of the second detected signals was not possible across all systems, and the weight of this potential bias cannot be estimated.

At the time of the study, none of the systems were able to detect early all events included in the gold-standard data set, substantiating the necessity for end-users to use several systems in parallel. The purpose of cross-feeding is to increase sensitivity by utilising all available pieces of information. However, such cross-feeding matters when several systems are used in parallel. In a previous study, some authors documented that major EI stakeholders routinely accessed four to seven different systems for event detection [7]. In this context, cross-feeding generates a substantial level of duplication for end-users. In the current study, 43% of signals detected by automated systems would have already been seen on a fellow system. This stresses the importance of developing a common tool that would combine system outputs and specificities while reducing duplication.

Overall 97% of detected signals were published in seven languages (English, Spanish, French, Russian, Portuguese, Arabic and Chinese), findings consistent with another study [16].These languages were the first integrated by the systems and as such the linguistic methodology (i.e. ontology) might be better developed for those languages than for languages incorporated more recently. Signals in all languages incorporated in the systems were systematically considered. Yet, it cannot be formally excluded that it might have been easier for analysts to detect information published in English, French, Spanish, or Portuguese than in information published in languages requiring systematic translation (e.g., Arabic, Chinese) or rarely used. Despite this potential bias, the results underline the importance of multiple languages tools.

The lack of recognised and consistently available reference sources across diseases and regions represents a major challenge to the evaluation of EI and biosurveillance systems [17], [18]. The choice of the gold-standard for this study (BHI) might have impacted the results. The disease and the location of occurrence are among the selection criteria used for EI at InVS. Events occurring in an area close to a French territory were more likely to be retained. Arboviruses represent a risk of exportation especially in overseas territories where competent vectors are present [19], as illustrated in 2006 by the outbreak of chikungunya virus, which affected over one third of the population of both Reunion and Mayotte islands [20]. For France, the circulation of major arthropod-borne infections in previously non-endemic areas is perceived as a threat to the blood supply, and therefore is a topic of high interest. This may partially explain both the high proportion (64%) of vector-borne diseases among reported events and the specific attention placed on those events. For instance, 2010 was a year marked by unprecedented WNV circulation in the Mediterranean area [21] and all 18 WNV infection events reported in 2010 occurred in Mediterranean countries not previously considered as endemic. Hence, other EI stakeholders, with different objectives or disease distribution, would certainly select different events resulting in another gold-standard data set. The performance of the EI process might also affect the results. Unfortunately, in the absence of a recognised international gold-standard, the sensitivity of the chosen gold-standard could not be assessed. This assessment should be carried out, but was beyond the current studies objectives. Although, these results are not fully representative, France’s large geographic distribution (spread over four continents) and systematic and stable approach to EI suggest that the results from this study provide a larger overview that can be transposed to other contexts. Others studies using other gold-standards should be implemented to better assess and ensure generalizability of the results.

The number of events included in the gold-standard was too limited to allow stratified analysis and may have undermined potential associations. For example, it is likely that some systems might have developed specific competences in specific languages, for certain geographic regions, or specific diseases, but this aspect could not be explored since biosurveillance systems are in constant evolution, and as such, the short time frame was chosen to limit intra-system variability. An extended study time period to generate more disease events could be considered for a future study.

The retrospective search for information might have influenced the results. In a previous study, authors found that prospective detection rates were 17% lower than the retrospective sensitivity rate [7]. However; this bias applies equally to all systems and does not affect the overall findings. Similarly, in this study no significant difference (p = 0.53) was found between the two InVS epidemiologists suggesting that the user-bias was limited.

In conclusion, infectious diseases, environmental issues, and potential bioterrorist threats will continue to pose major risks for global health security and epidemic intelligence is now an essential component of early warning systems. Overall, the systems’ disease detection capabilities are complementary (synergistic) with demonstrated timeliness and sensitivity [7]. The output from these systems and also the expertise of the public health institutions responsible for EI should be pooled for optimal early detection. Internet biosurveillance systems have evolved substantially; sufficient data is now available to implement robust validation studies using epidemiological approaches against an official comparison data set. Moreover, larger scope studies should be implemented that would prospectively involve major stakeholders, increase the number of epidemiologists involved, and enable implementation of innovative strategies to pool the expertise developed by the different systems. Especially, a more robust composite gold-standard that pools information and expertise from national and international institutions in charge of EI activities should be developed.
